# Effects of Osmotic and Hydrostatic Pressures on Milk and Antimicrobial Substance Production in Lactating Goats

**DOI:** 10.1111/asj.70199

**Published:** 2026-06-10

**Authors:** Donghyeon Kang, Jirapat Jaisue, Takahiro Nii, Ken Kobayashi, Naoki Isobe

**Affiliations:** ^1^ Graduate School of Integrated Sciences for Life Hiroshima University Higashi‐Hiroshima Japan; ^2^ Research Faculty of Agriculture Hokkaido University Sapporo Japan

**Keywords:** antimicrobial substance, goat, hydrostatic pressure, milk, osmolarity

## Abstract

Osmotic and hydrostatic (intramammary pressure) pressures within the mammary gland may stimulate mammary epithelial cells and leukocytes, which alter milk production and immune functions. We aimed to clarify the effects of different volumes and concentrations of NaCl solution on milk and antimicrobial substance production in lactating goats. Intramammary infusions of different NaCl solution concentrations (0.9% and 2.7%) at different volumes (50 mL or until full in the udder) were carried out; milk samples were collected for 5 days after infusion. The infusions resulted in significant daily effects on milk yield, somatic cell count (SCC), sodium ion (Na^+^) and potassium ion (K^+^), milk amyloid A (MAA), S100A7, and lactoferrin (LF) concentrations. Sodium and potassium ions, SCC, and MAA levels differed depending on the infused volume, whereas those of S100A7 and LF differed according to the NaCl concentration. These results suggest that short‐term increases in hydrostatic and osmotic pressures modulate milk production and stimulate antimicrobial factor production in the mammary glands. Hence, they highlight a potential approach toward enhancing innate immune function in the udder.

## 1 Introduction

In the mammary gland, mammary epithelial cells (MECs) surround the alveolus, synthesize and secrete milk into the alveolar lumen, and their milk is discharged into the milk ducts (Jaswal et al. 2022). MECs are exposed to physical stimuli such as hydrostatic pressure caused by milk accumulation immediately before milking and osmotic pressure due to changes in milk components resulting from excessive milk accumulation after the start of dry‐off (Fleet and Peaker 1978; Katthöfer et al. 2024). Kobayashi et al. (2023) reported that applying high hydrostatic pressure to mouse MECs for 8 h in vitro decreased the expression of β‐casein, a major milk protein, and increased the expression of claudin‐4, a tight junction protein. These results suggest that milk production may be regulated by hydrostatic and osmotic pressures (Kobayashi et al. 2023).

Mastitis results in reduced milk production (Seegers et al. 2003). In clinical mastitis, disruption of the blood–milk barrier may facilitate the transfer of blood‐derived components into milk, potentially altering the osmotic environment within the mammary gland (Kandeel et al. 2019). Furthermore, in vitro studies have suggested that sustained high hydrostatic pressure may inactivate signaling pathways associated with tight junction formation and adversely affect tight junction integrity (Kobayashi et al. 2023). However, evidence directly linking hydrostatic pressure to mastitis remains limited. Although antibiotics are widely used to treat mastitis, concerns remain regarding the suspension of milk shipments and emergence of drug‐resistant bacteria (Isobe 2017). Therefore, establishing countermeasures against mastitis by maximizing the immune function in cows is essential. The mammary glands possess innate and adaptive immunity. Innate immunity consists of leukocytes and antimicrobial peptides with a broad antibacterial spectrum (Zhang et al. 2014; Isobe 2017).

Various cellular sensors have been reported to detect physical stimuli such as changes in osmolarity, temperature, and mechanical stress, and these sensors may contribute to the regulation of mammary gland function. Temperature‐sensitive sensors include Transient Receptor Potential (TRP) channels such as Transient Receptor Potential Vanilloid 1 (TRPV1) and Transient Receptor Potential Ankyrin 1 (TRPA1), whereas osmolarity‐sensitive systems include Piezo Type Mechanosensitive Ion Channel Component 1 (Piezo1) and the Transcription Factor Nuclear Factor of Activated T‐cells 5 (NFAT5) (Liao et al. 2026; Xiao 2024; Halterman et al. 2012). Among these sensors, Transient Receptor Potential Vanilloid 4 (TRPV4) is notable in that it responds to multiple types of physical stimuli, including both temperature and osmotic changes, and is expressed on the membrane of MECs (Shibasaki 2016). Islam et al. (2020) reported that β‐casein expression increased in mouse mammary tissue cultured at 39°C for 72 h, compared with tissue cultured at 37°C for 72 h. Furthermore, Tsugami et al. (2021) demonstrated that local heat treatment of goat udders increased somatic cell count (SCC) and IgA concentration in milk. These findings suggest that physical stimuli can modulate milk production and immune‐related responses in the mammary gland. However, the effects of hydrostatic and osmotic pressure on the immunological function of the mammary glands remain unclear.

Antimicrobial peptides play a crucial role in mammary gland defense. S100A7 is a calcium‐binding protein that exhibits antimicrobial activity against *Escherichia coli* in humans and cows (Regenhard et al. 2009). In goats, S100A7 mRNA is expressed mainly in the teat skin; intramammary infusion of lipopolysaccharide (LPS) significantly increases milk S100A7 concentrations 24 h after infusion (Zhang et al. 2014; Isobe 2017). Lactoferrin (LF) is an iron‐binding glycoprotein that suppresses bacterial growth owing to its high iron‐binding affinity (Orsi 2004). In cows, intramammary infusion of LPS significantly increases LF concentration (Hyvönen et al. 2010). These findings indicate that antimicrobial peptides are produced and regulated in response to bacterial infection in the mammary gland. Hence, these antimicrobial peptides are crucial for mammary gland defense. Therefore, promoting the production of antimicrobial peptides is considered effective in enhancing the defense system against mastitis. Recent studies suggest that S100A7 expression in the mammary gland may be regulated by mechanosensitive and osmotic stress–responsive pathways, potentially involving TRPV4, thereby linking physical stimuli to antimicrobial defense responses (Bhatt et al. 2019; Guo et al. 2023).

In addition to antimicrobial components such as S100A7 and lactoferrin, inflammatory markers also provide important information regarding the inflammatory response in the mammary gland. Milk amyloid A (MAA) is an acute‐phase protein that is markedly elevated in milk during mastitis and is widely recognized as a biomarker of mammary inflammation (Webb 2021). Therefore, in this study, MAA was used to assess inflammatory responses to osmotic stimulation, enabling a comprehensive evaluation of host defense responses.

Previous studies have reported that TRPV4 promotes the expression of antimicrobial peptides and inflammatory cytokines via activation of the p38 MAPK signaling pathway (Saklatvala 2004). Hence, TRPV4 activation by osmotic stimulation may be involved in the production of antimicrobial substances and milk by MECs, although this remains unclear. Elucidating this mechanism is important for understanding how physical stimuli regulate mammary gland function and innate immune responses. It may also lead to the development of simple, cost‐effective, and readily applicable mastitis control strategies, such as the potential use of NaCl solutions as a practical therapeutic option. Therefore, we hypothesized that increased osmotic and hydrostatic pressures in the mammary gland reduce milk production, thereby increasing the concentration of antimicrobial substances and enhancing local antimicrobial capacity. We aimed to clarify the effects of osmotic and hydrostatic pressures in the mammary glands of lactating goats on the production of milk and antimicrobial substances in these glands.

## 2 Materials and Methods

### 2.1 Experimental Animals

In this study, 35 mammary halves of 16 lactating Tokara goats (milk yield: 32–350 mL/day; body weight: 20–25 kg; parity: 1–4) were used. The goats were housed individually and fed 0.6 kg hay cubes and 0.2 kg rolled barley per day. Water and mineral salt blocks were provided ad libitum. The amount of feed was calculated based on the Japanese feeding standard for sheep reported by Jaisue et al. (2025).

All animal experiments were approved by the Animal Care and Use Committee of Hiroshima University (Approval No.: C14‐5) and conducted in accordance with the university's animal experimentation guidelines.

### 2.2 Intramammary Infusion of NaCl Solution

Goats were divided into four groups based on NaCl infusion. Different concentrations of NaCl solution (0.9% [×1] and 2.7% [×3]) were infused into the mammary glands; infusion quantities varied from 50 mL or until full in the udder (100–800 mL) as ×1–50 (*n* = 11), ×3–50 (*n* = 12), ×1‐full (*n* = 6), ×3‐full (*n* = 6).

### 2.3 Collection of Milk Samples

The day of NaCl solution infusion was defined as Day 0; milk samples were collected once daily for 6 days consecutively (Days 0–5). Milk samples were collected once daily by hand‐milking from halves. Only the udder half with SCC ≤ 1,000,000 cells/mL was used for the experiment.

After measuring milk yield, the collected milk samples were centrifuged at 2300 ×*g* for 5 min at 4°C to remove milk fat. The resulting skim milk was stored at −30°C for the measurement of sodium ion (Na^+^) and potassium ion (K^+^) concentrations, which were measured using the compact water quality meter LAQUA twin‐Na‐11 (HORIBA Scientific, Kyoto, Japan) and LAQUA twin‐K‐11 (HORIBA Scientific, Kyoto, Japan) (Ohno et al. 2023), and antimicrobial substances concentration.

The somatic cell pellets obtained by centrifugation were suspended in 2 mL phosphate‐buffered saline (PBS) and centrifuged twice at 65 ×*g* for 5 min at 4°C for washing. After supernatant removal, the pellet was resuspended in PBS; SCC was measured using the Countess II FL Automated Cell Counter (Thermo Fisher Scientific, MA, United States).

### 2.4 ELISA

S100A7, LF, and MAA concentrations were measured using competitive ELISA, according to previously reported methods (Kuwahara et al. 2017; Zhang et al. 2014; Marufah et al. 2025). S100A7 and MAA antibodies were produced by immunizing rabbits with the S100A7 (CFEKQDKNKDRKID) and MAA (CREANYKGADKYFHARGNYD) peptides. The LF antibody was purchased from Life Laboratory Company (LL‐A0013, Yamagata, Japan).

### 2.5 Statistical Analysis

To compare milk yield, SCC, and concentrations of Na^+^, K^+^, LF, S100A7, and MAA in milk, the JMP Pro 18 (SAS Institute Inc., NC, United States) software was used. A linear mixed‐effects model was applied, with the infused amounts, concentrations, days, and their interaction included as fixed effects, and individual goats included as a random effect to account for repeated measurements within each goat. The Wilcoxon signed‐rank test was used to compare the differences between treatment groups and the daily differences from Day 0 within each group. All data are presented as means ± standard error of the mean (SEM). Statistical significance was set at *p* < 0.05. Milk yield, LF, S100A7, and MAA data are presented as ratios relative to values in Day 0 to account for inter‐individual variability. Transformation to log did not alter normality or statistical conclusions; therefore, non‐transformed ratio data are shown.

## 3 Results

Intramammary infusion of NaCl solution resulted in significant differences in the day for all measured variables (Table 1). Additionally, the milk components, Na^+^ and K^+^, and the inflammation markers, SCC and MAA, exhibited significant differences depending on the infused solution volume. Furthermore, concentrations of the antimicrobial substances, S100A7 and LF, differed significantly according to the infused solution concentration.

**TABLE 1:** Results of three‐way mixed‐effect model analysis for milk yield and components following intramammary infusion of saline.

	Infusion^a^					*p* ^b^					
	50 mL		Full								
Item	×1	×3	×1	×3	SEM	D	C	V	D × C	D × V	C × V
Milk (mL)	179	131	100	119	5.26	< 0.01	0.270	0.372	0.091	< 0.01	0.511
Na^+^ (mg/L)	428a	577b	913ab	939ab	39.5	< 0.01	0.018	< 0.01	0.016	< 0.01	0.610
K^+^ (mg/L)	1857	1899	1515	1619	27.0	< 0.01	0.568	< 0.01	0.819	< 0.01	0.642
log10 SCC (cell/mL)	5.62a	6.02b	6.51ab	6.41ab	0.05	< 0.01	< 0.01	< 0.01	0.517	< 0.01	< 0.01
MAA (μg/mL)	1.08	1.36	220	86	0.07	< 0.01	0.427	< 0.01	0.556	< 0.01	0.957
S100A7 (μg/mL)	1.27	1.36	4.71	4.32	0.19	< 0.01	0.018	0.188	< 0.01	0.149	0.092
LF (μg/mL)	67.6a	155b	291ab	489ab	125	< 0.01	0.016	0.405	0.198	0.263	0.061

*Note:* Values with different letters (a, b) show significant differences between groups (*p* < 0.05).

Abbreviations: LF: lactoferrin; MAA: milk amyloid A; SCC: somatic cell count; SEM: ± standard error of the mean.

^a^ NaCl solution 0.9% (×1) and 2.7% (×3) was infused 50 mL, or until the udder was filled (Full).

^b^ D, day after infusion; C, concentration of NaCl solution; V, volume of infused solution.

**FIGURE 1:**
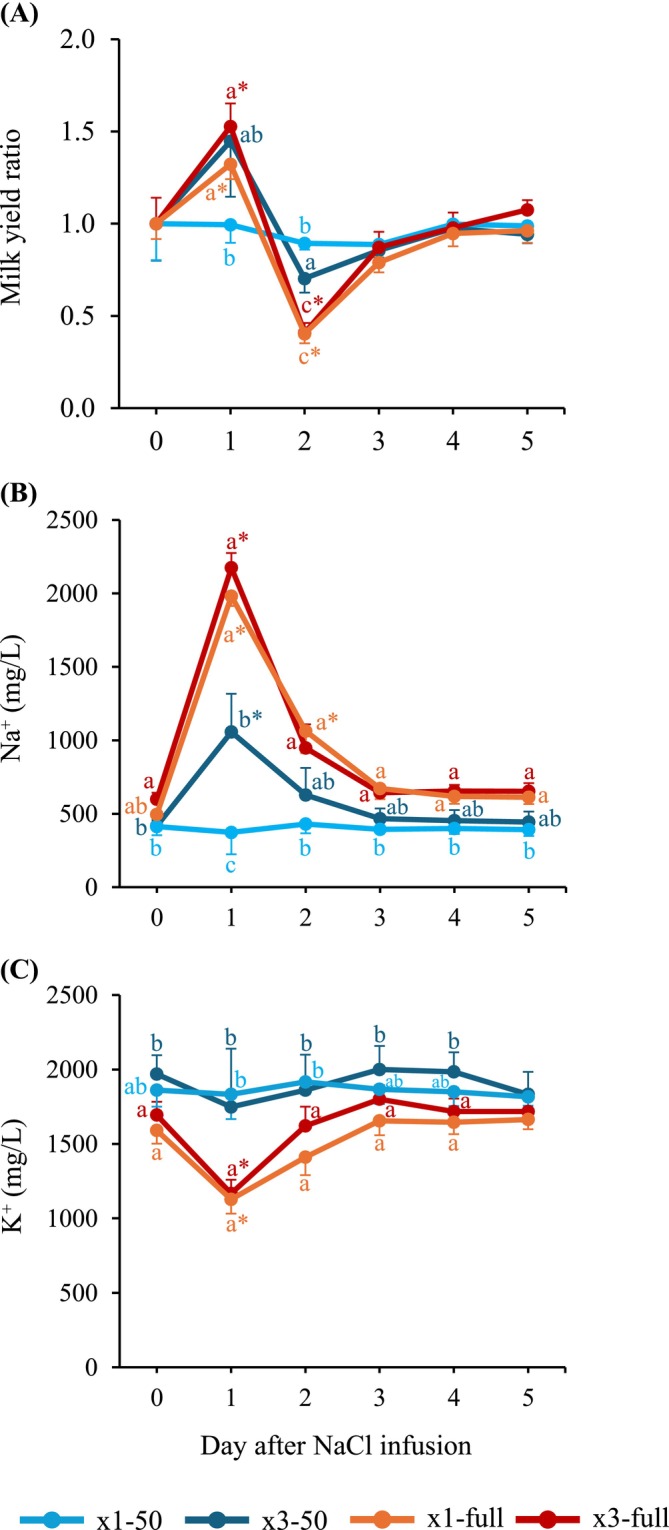
Effects of intramammary osmotic pressure with and without high hydrostatic pressure on milk yield (A), Na^+^ (B), and K^+^ (C) in goats. Goats received intramammary infusions of either 0.9% NaCl, 50 mL (×1–50; *n* = 11), 0.9% NaCl full volume (×1‐full; *n* = 12), 2.7% NaCl, 50 mL (×3–50; *n* = 6), or 2.7% NaCl full volume (×3‐full; *n* = 6). Results are presented as mean ± standard error of the mean (SEM). Values with different letters (a, b, c) indicate significant differences between groups (*p* < 0.05). Asterisks (*) indicate significant differences in comparison to Day 0 (*p* < 0.05).

In all groups, except the ×1–50 group, milk yield increased on Day 1 and decreased on Day 2, whereas Na^+^ concentration increased significantly on Day 1 and subsequently recovered (Figure 1A,B). Potassium ion concentration decreased significantly on Day 1 in the ×1‐ and ×3‐full groups, whereas no changes were observed in either the ×1‐ or ×3–50 groups (Figure 1C).

**FIGURE 2:**
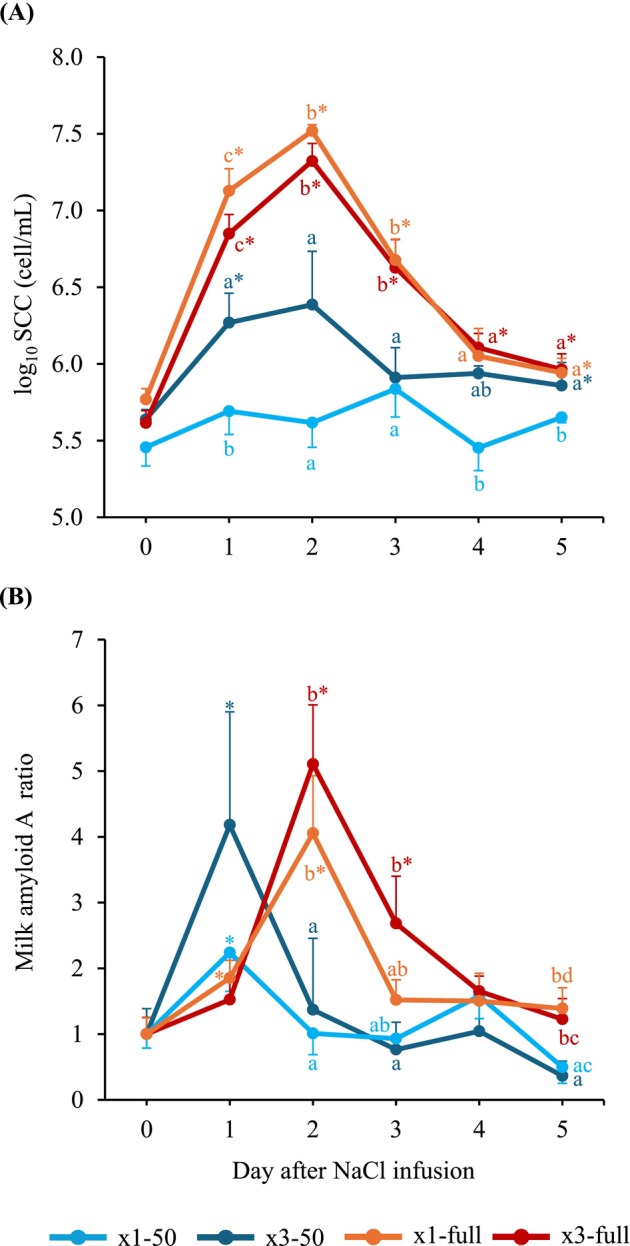
Effects of intramammary osmotic pressure with and without high hydrostatic pressure on somatic cell count (A), and milk amyloid A (B) in goats. Goats received intramammary infusions of either 0.9% NaCl, 50 mL (×1–50; *n* = 11), 0.9% NaCl full volume (×1‐full; *n* = 12), 2.7% NaCl, 50 mL (×3–50; *n* = 6), or 2.7% NaCl full volume (×3‐full; *n* = 6). Results are presented as mean ± standard error of the mean (SEM). Values with different letters (a, b, c, d) indicate significant differences between groups (*p* < 0.05). Asterisks (*) indicate significant differences in comparison to Day 0 (*p* < 0.05).

SCC increased significantly in all groups except the ×1–50 group; in particular, high values were maintained until Day 3 in the x1‐ and ×3‐full groups (Figure 2A). MAA significantly increased in all groups; the ×1‐ and ×3–50 groups exhibited a significant increase, peaking on Day 1, whereas the ×1‐ and ×3‐full groups also exhibited a significant increase, peaking on Day 2 (Figure 2B).

**FIGURE 3:**
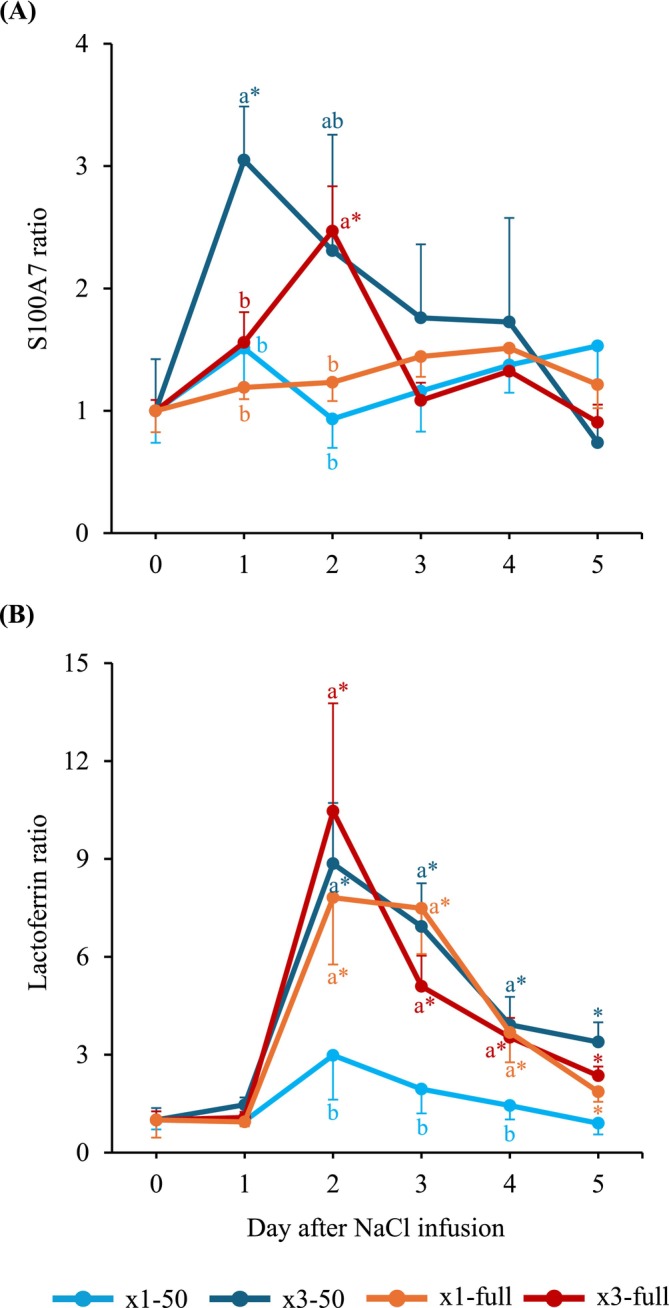
Effects of intramammary osmotic pressure with and without high hydrostatic pressure on S100A7 (A), and lactoferrin (B) in goats. Goats received intramammary infusions of either 0.9% NaCl, 50 mL (×1–50; *n* = 11), 0.9% NaCl full volume (×1‐full; *n* = 12), 2.7% NaCl, 50 mL (×3–50; *n* = 6), or 2.7% NaCl full volume (×3‐full; *n* = 6). Results are presented as mean ± standard error of the mean (SEM). Values with different letters (a, b) indicate significant differences between groups (*p* < 0.05). Asterisks (*) indicate significant differences in comparison to Day 0 (*p* < 0.05).

S100A7 expression increased significantly in the ×3–50 group on Day 1 and in the ×3‐full group on Day 2, whereas no significant increase was found in both ×1 groups (Figure 3A). Lactoferrin increased significantly in all groups except the ×1–50 group and remained at high levels until Day 5 (Figure 3B).

## 4 Discussion

Hydrostatic pressure may temporarily suppress milk production. In the present study, we investigated the effects of intramammary osmotic pressure with and without hydrostatic conditions on milk and antimicrobial substance production in the udder. Milk yield decreased significantly on Day 2 in the ×1‐full and ×3‐full groups, whereas no significant decrease was observed in the ×1–50 and ×3–50 groups. Previous studies have reported that hydrostatic pressure can activate TRPV4, which may promote inflammatory responses via pathways such as JAK2/STAT3 and affect MEC function (Li et al. 2021; Kobayashi et al. 2023). Additionally, high intramammary pressure by milk accumulation after the cessation of milking decreases milk yield (Peaker 1980). Furthermore, excessive milk accumulation immediately after the tentative cessation of milking reduces milk yield (Purba et al. 2021). Kobayashi et al. (2023) reported that high hydrostatic pressure for 1 h in mouse MECs activated p38, which subsequently induced STAT5 (a key protein for milk production) inactivation, suggesting that high hydrostatic pressure reduced milk production ability. However, the underlying molecular mechanisms, including the potential involvement of TRPV4‐related signaling pathways, were not directly examined in this study. Therefore, the decrease in milk yield observed in this study may be caused by the impaired milk production capacity of MECs, which is caused by an increase in intramammary pressure under high hydrostatic pressure. A limitation of this study is that milk components (e.g., lactose, β‐casein) were not analyzed; therefore, it can at least be concluded that milk volume changed.

Intramammary infusion of NaCl altered ionic balance within the mammary gland. Regarding ion concentrations, the infusions of ×1 and ×3 NaCl solutions resulted in a high‐Na^+^ environment within the udder, causing a significant increase in Na^+^ concentration and a significant decrease in K^+^ concentration. Sodium ion concentration in saline (0.9%) is substantially higher than that in milk (0.036%; Stergiadis et al. 2019). Potassium ion concentration decreases as milk Na^+^ concentration increases (Stelwagen et al. 1999). On their cell membranes, MECs express high levels of Na^+^/K^+^‐ATPase, which regulates intracellular Na^+^ and K^+^ concentrations (Rajasekaran et al. 2001). Under normal conditions, Na^+^‐dependent transporters maintain low intracellular Na^+^ levels, which is an important environment for the synthesis of milk components, including lactose, in MECs (Shennan and Peaker 2000; Contreras et al. 2024).

High hydrostatic and osmotic pressures may induce inflammatory responses in the mammary gland. Sodium ion concentration remained high in the ×1‐full and ×3‐full groups on Day 2. Mastitis increases Na^+^ concentration in milk (El Zubeir et al. 2005). The high SCC on Day 2 suggested that high Na^+^ levels depended on mammary gland inflammation. Regarding inflammatory markers, SCC and MAA increased significantly in the full and ×3–50 groups, suggesting that high hydrostatic and osmotic pressures can induce inflammation in the mammary glands. Serum amyloid A is primarily synthesized in the liver during systemic acute inflammation, although local production in MECs and other tissues, such as the small intestine and stomach, has also been reported to cause local inflammation (Urieli‐Shoval et al. 2000; Upragarin et al. 2005; Webb 2021). In the present study, MAA levels increased with those of SCC. Therefore, MAA in milk may be a reliable indicator of mastitis.

Lactoferrin increased significantly in the ×3–50 group and the ×1‐ and ×3‐full groups. These changes are similar to those observed of SCC and MAA. Lactoferrin is normally produced by MECs; however, neutrophils are a major source of LF under inflammatory conditions (Isobe 2017). Hence, the increase in LF levels observed in this study is likely attributable to leukocyte‐derived LF following inflammation. These changes may reflect an inflammation‐associated response rather than a physiological enhancement of innate immune function.

Osmotic pressure may play a key role in regulating S100A7 production. S100A7 increased only in the ×3–50 and ×3‐full groups but not in the ×1–50 and ×1‐full groups. This finding indicates that osmotic pressure is the main factor in promoting S100A7 production. S100A7 is synthesized by MECs (Zhang et al. 2014). MECs express TRPV4 channels, which are activated by cell swelling or shrinkage in response to osmotic mechanical stimuli (White et al. 2016; Liedtke and Friedman 2003). Previous studies have suggested that TRPV4 activation can promote S100A7 production via pathways such as p38/MAPK (Bhatt et al. 2019; Guo et al. 2023). Therefore, TRPV4‐related signaling may be involved in the increase in S100A7 observed in this study. However, as these signaling pathways were not directly assessed, this interpretation should be considered speculative. In addition, given that inflammatory responses were also induced in this study, as indicated by increased SCC and MAA, the observed increase in S100A7 may reflect both inflammation‐associated and osmotic stimulus–dependent mechanisms, suggesting that antimicrobial substance production is regulated by both processes.

In conclusion, the findings of this study suggest that high hydrostatic and osmotic pressures for 1 day modulate milk production and stimulate antimicrobial factor production in the mammary glands. These insights highlight the potential of enhancing innate immune function in the udder and may contribute to the development of novel strategies for mastitis control. In the present study, the infusion of physiological saline into the mammary gland, followed by retention for 24 h, induced a transient inflammatory response. Conversely, under field conditions in dairy practice, infused solutions are typically expelled immediately after administration (Shinozuka et al. 2009). Thus, the prolonged retention time (24 h) used in this study is not fully representative of field conditions, and its direct applicability to practical dairy management may be limited. Therefore, it is necessary to investigate whether similar inflammatory changes occur when the infused solution is discharged promptly. Additionally, NaCl solutions at higher concentrations (7.2%) are sometimes infused in the field (Kikuchi et al.^2019^); hence, it would be of interest to examine the inflammatory responses induced by transient infusion of NaCl solutions at concentrations higher than ×3 physiological saline.

## Funding

This work was supported by Japan Society for the Promotion of Science, 10.13039/501100001691, 24K01903.

## Conflicts of Interest

The authors declare no conflicts of interest.

## Data Availability

The data that support the findings of this study are available on request from the corresponding author. The data are not publicly available due to privacy or ethical restrictions.
